# Sydenham Society

**Published:** 1845-06

**Authors:** 


### BIBLIOGRAPHICAL NOTICES.

SYDENHAM SOCIETY. *The seven books of Paulus Ægineta. Translated from the Greek, with a Commentary embracing a complete view of the knowledge possessed by the Greeks, Romans, and Arabians on all subjects connected with Medicine and Surgery.* By FRANCIS ADAMS. In three volumes. Vol. I., 8vo. pp. 683; London, 1844.

